# A Place-Based Community Health Worker Program: Feasibility and Early Outcomes, New York City, 2015

**DOI:** 10.1016/j.amepre.2016.08.034

**Published:** 2017-03

**Authors:** Priscilla M. Lopez, Nadia Islam, Alexis Feinberg, Christa Myers, Lois Seidl, Elizabeth Drackett, Lindsey Riley, Andrea Mata, Juan Pinzon, Elisabeth Benjamin, Katarzyna Wyka, Rachel Dannefer, Javier Lopez, Chau Trinh-Shevrin, Karen Aletha Maybank, Lorna E. Thorpe

**Affiliations:** 1 City University of New York School of Public Health and Health Policy, NYU-CUNY Prevention Research Center, New York, New York; 2 New York University School of Medicine, NYU-CUNY Prevention Research Center, New York, New York; 3 New York City Department of Health and Mental Hygiene, Long Island City, New York; 4 New York City Housing Authority, New York, New York; 5 Community Services Society, New York, New York

## Abstract

**Introduction:**

This study examined feasibility of a place-based community health worker (CHW) and health advocate (HA) initiative in five public housing developments selected for high chronic disease burden and described early outcomes.

**Methods:**

This intervention was informed by a mixed-method needs assessment performed December 2014–January 2015 (representative telephone survey, *n*=1,663; six focus groups, *n*=55). Evaluation design was a non-randomized, controlled quasi-experiment. Intake and 3-month follow-up data were collected February–December 2015 (follow-up response rate, 93%) on 224 intervention and 176 comparison participants, and analyzed in 2016. All participants self-reported diagnoses of hypertension, diabetes, or asthma. The intervention consisted of chronic disease self-management and goal setting through six individual CHW-led health coaching sessions, instrumental support, and facilitated access to insurance/clinical care navigation from community-based HAs. Feasibility measures included CHW service satisfaction and successful goal setting. Preliminary outcomes included clinical measures (blood pressure, BMI); disease management behaviors and self-efficacy; and preventive behaviors (physical activity).

**Results:**

At the 3-month follow-up, nearly all intervention participants reported high satisfaction with their CHW (90%) and HA (76%). Intervention participants showed significant improvements in self-reported physical activity (*p*=0.005) and, among hypertensive participants, self-reported routine blood pressure self-monitoring (*p*=0.013) compared with comparison participants. No improvements were observed in self-efficacy or clinical measures at the 3-month follow-up.

**Conclusions:**

Housing-based initiatives involving CHW and HA teams are acceptable to public housing residents and can be effectively implemented to achieve rapid improvements in physical activity and chronic disease self-management. At 3-month assessment, additional time and efforts are required to improve clinical outcomes.

## Introduction

Place-based initiatives are a potentially effective approach to reduce health disparities among residents living in underserved neighborhoods.^1^ Community health workers (CHWs) and health advocates (HAs) can play a role in advancing community health.^2^ CHWs are health professionals who provide healthcare support and have a close understanding of communities they serve through shared ethnicity, culture, language, and life experiences.^3^ HAs provide health insurance enrollment and post-enrollment healthcare navigational assistance.^4^ In limited settings, CHWs have been deployed in public housing to address specific health needs, support health promotion, or build social capital.^5–8^ None have been launched with municipal funds.

In January 2015, a partnership among a city health agency, housing authority, community-based organizations, and academic partners was launched to address the health of residents in East Harlem, New York City, a neighborhood with high rates of obesity, diabetes, and barriers to health care.^9^ This publicly financed initiative, the Harlem Health Advocacy Partnership, was guided by a health equity framework^10^ and offered CHW services to housing residents to manage chronic diseases and set health goals, as well as insurance navigational assistance by a team of HAs to help residents find, understand, and use affordable/low-cost health insurance and health care, and review plan options. This study aimed to demonstrate feasibility and examine preliminary effectiveness.

## Methods

### Study Design

Intervention design and protocol was developed between June 2014 and January 2015 through meetings between partner institutions and interactions with public housing resident leaders. Participants were recruited from five public housing developments representing 12,720 residents; developments were selected for high hemoglobin A1c levels per health surveillance data.^11^ Intervention inclusion criteria were age ≥ 18 years; self-reported diagnosis of hypertension, diabetes, or asthma; fluency in English or Spanish; and participation consent.

The intervention was informed by a mixed-method needs assessment performed by academic partners from December 2014 to January 2015 (random sample telephone survey, *n*=1,663; six focus groups, *n*=55) among residents living in selected housing developments, as well as in five nearby developments with comparable demographic/health status make-up (comparison community). Needs assessment details have been published elsewhere as a report to policymakers and stakeholders.^11^

Intervention evaluation design was a non-randomized, controlled quasi-experiment. Most intervention and all comparison participants were recruited from the needs-assessment telephone survey if they reported hypertension, diabetes, or asthma diagnoses and expressed interest; additional recruitment of intervention participants occurred via local health fairs and outreach. Intake and 3-month follow-up data collection by academic partners occurred February–December 2015; a brief in-person questionnaire and biometric assessment of blood pressure, height, and weight were administered at each time point. All participants received a $20 cash incentive for completing follow-up survey.

CHWs were recruited from targeted housing residences and broader East Harlem community, hired by a local community- based organization, and trained in CHW core competencies, health education, goal setting, and in facilitating linkages to care by referring to HAs employed by another local community-based organization expert in health insurance enrollment and access to care issues. The CHW intervention included six or more educational/instrumental support visits, as well as referral to HAs as needed. CHW and HAs were trained separately on respective competencies but jointly on Harlem Health Advocacy Partnership protocol/referral processes. HA support was available to both intervention and comparison communities.

### Measures

At each visit, blood pressure was measured three times and averaged for analyses. Self-reported physical activity, general mental health status, self-perceived chronic disease management, healthcare access, self-efficacy, and quality of life were assessed at each time point.

### Statistical Analysis

Group differences in demographics, health insurance, and general health characteristics were compared using chi-square tests. Between-group differences in changes in outcome measures from baseline to follow-up were assessed using mixed models for continuous outcomes and generalized estimating equations for categorical outcomes. Each model included time (baseline, follow up); group (intervention, comparison); and their interaction term. Models adjusted for baseline age to account for older average age among intervention participants and conducted using SAS, version 9.2, or Stata, version 12. Analyses were conducted in 2016.

## Results

Needs assessment survey results confirmed no statistically significant differences in aggregate demographics, health insurance status/type, self-reported health, or health behaviors between residents in intervention and comparison developments (Appendix, available online). Prevalence of targeted health conditions (hypertension, diabetes, and asthma) was also similar.

**Figure 1:**
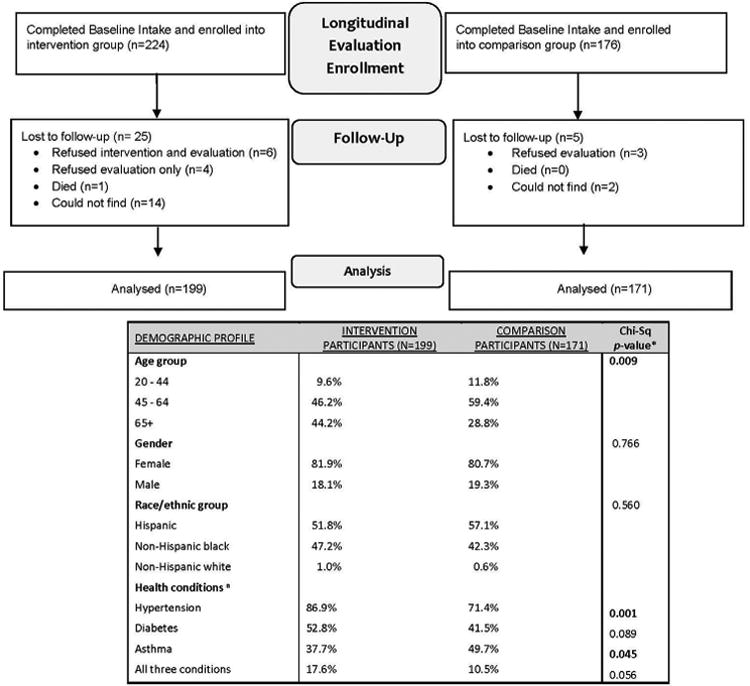
Participant CONSORT diagram for longitudinal evaluation of HHAP intervention, East Harlem, New York City, 2015. *Note:* Boldface indicates statistical significance (*p*<0.05). ^a^Health conditions are adjusted for age. HHAP, Harlem Health Advocacy Partnership.

Despite comparable aggregate demographic/health status profiles between residents in intervention and comparison developments, participants who enrolled into the intervention were significantly older than those enrolled in the comparison group (Figure 1). A greater proportion of intervention participants self-reported a hypertension diagnosis (87% vs 71%, *p*=0.001) and being diagnosed with all three reported conditions (18% vs 11%, *p*=0.056), but a lower proportion reported having diagnosed asthma (38% vs 50%, *p*=0.045). The intervention group experienced greater attrition (11%) between baseline and follow-up than the comparison group (3%), with a total response rate of 93%. Analyses were based on 199 intervention and 171 comparison participants with follow-up information, adjusting for between-group age differences.

Nearly all (90%) intervention participants reported high satisfaction with their CHW and most (76%) established personal goals at follow-up. Measured clinical outcomes did not improve in intervention versus comparison participants over time (Table 1). At follow-up, however, intervention participants reported greater improvements in physical activity than comparison participants (*p*=0.005), and those with hypertension reported greater improvements in self-monitoring of blood pressure (*p*=0.013). Intervention participants were also more likely to receive help from an HA in solving health insurance problems (p=0.019, not shown). Of those receiving HA support, 97% found services helpful. Compared with comparison participants, intervention participants were more likely to report at follow-up having changed their health insurance or insurance status (11% vs 4%, *p*=0.009), and to report having changed their personal doctor (14% vs 6%, *p*=0.024). Open-ended responses confirmed high satisfaction with the program (Appendix, available online).

## Discussion

This evaluation of a publicly funded, place-based CHW initiative found using locally recruited CHWs and facilitated referrals to HAs to be well received by low-income housing residents and effective at rapidly improving services navigation, self-reported physical activity, and self-management behaviors. Findings are consistent with literature suggesting that CHW programs generally achieve positive outcomes for chronic disease prevention and self-management when supportive relationships with patients are developed,^7,12,13^ and high satisfaction levels and risk reduction can be achieved when programs established in public housing settings use residents as workers.^5,6^ In early intervention months, it was challenging to improve clinical outcomes. Other studies have documented challenges in improving clinical outcomes, depending heavily on integration with clinical services, dose, and intervention standardization.^14,15^

### Limitations

Findings should be interpreted while being mindful of key limitations. First, this was a controlled quasi-experiment; participants in treatment and control groups were not randomly selected. Intervention participants were older and in worse health than comparison participants, reducing the effectiveness of the comparison proxy group. Second, sample size limited the statistical power to detect differences.

## Conclusions

These findings demonstrate the feasibility of a municipal health department leading a place-based CHW/HA intervention targeting multiple chronic conditions using public funds, catalyzed by multisector partnerships. Findings also stress the importance of collective monitoring of early results, which, for this project, has yielded increased attention to building formal clinical integration mechanisms and improved documentation of intervention fidelity. Such innovative models inform and align with Medicaid Expansion and other policy efforts designed to more effectively link communities to care.

## Supplementary Material

Supplemental file

## Figures and Tables

**Table 1:** Behavioral and Clinical Outcomes at Baseline and at 3-Month Follow-Up, Intervention Versus Comparison Group: East Harlem, New York City, 2015

	Intervention group (*n*=199)			Comparison group (*n*=171)			Estimated between-group difference	
Outcome	Baseline	3-month FU	*p*-value	Baseline	3-month FU	*p*-value	Difference (95% CI)	*p*-value
Measured clinical outcomes, M (SD)								
Systolic blood pressure (SBP)	132.8 (21.3)	133.0 (22.1)	0.866	128.8 (20.8)	124.3 (19.4)	**0.004**	3.79(-0.14, 7.72)	0.059
Diastolic blood pressure (DBP)	82.6 (12.3)	83.9 (13.7)	0.274	82.4 (12.3)	81.1 (12.5)	0.106	2.32(-0.05, 4.69)	0.055
BMI	33.5 (7.8)	33.7 (8.2)	0.415	34.3 (8.6)	34.1 (8.2)	0.102	0.39(-0.05, 0.82)	0.082
Self-reported behaviors, M (SD)								
Average number of days of physical activity, past 2 weeks	4.6 (5.1)	6.6 (5.8)	**<0.001**	5.7 (5.6)	5.9 (5.6)	0.840	1.90(0.58, 3.23)	**0.005**
General health								
% reporting mental health status as “Excellent,” “Very good,” or “Good”	56.7	69.0	**0.025**	70.0	66.5	0.972	0.12(-0.10, 0.34)	0.303
Hypertension management								
% reporting they are managing their hypertension well	80.5	88.8	**0.019**	85.1	86.7	0.781	0.07(-0.03, 0.17)	0.149
% with diagnosed hypertension at baseline who routinely measure their own blood pressure	45.6	60.7	**0.001**	44.3	43.8	0.897	0.15(0.03, 0.26)	**0.013**
Diabetes management								
% reporting they are managing their diabetes well	65.7	80.0	**0.011**	75.0	84.5	0.073	0.05(-0.09, 0.18)	0.504
% with diagnosed diabetes at baseline who routinely measure their glycemic levels	74.0	72.1	0.662	63.2	59.2	0.561	0.02(-0.12, 0.16)	0.819
Asthma management								
% reporting they are managing their asthma well	87.5	79.7	0.151	85.0	82.4	0.589	-0.05(-0.19, 0.09)	0.491

*Note:* Boldface indicates statistical significance (*p*<0.05). Absolute difference is shown for blood pressure, BMI, and average days of physical activity in the estimated between-group difference column. Difference in % improved is shown for percentage-based outcomes.

FU, follow-up.
