# Supplementary material for: A Place-Based Community Health Worker Program: Feasibility and Early Outcomes, New York City, 2015
Source: Am J Prev Med. Author manuscript; Available in PMC 2017 Oct 25. (PMC5656273; doi:10.1016/j.amepre.2016.08.034)
Supplement: Supplemental file [file NIHMS874748-supplement-Supplemental_file.pdf]

Appendix Table 1. Pre-Intervention Rapid Needs Assessment Survey Results from Intervention and Comparison Public Housing Developments and Related Focus Group Findings: December 2014-January 2015, East Harlem, New York City

	**Intervention developments**		**Comparison developments**		***p*-value**
**Characteristic**	**Weighted N**	**%**	**Weighted N**	**%**	
Total	6,241		8,356		
**I. Demographics**					
Age group					0.720
35-49	1,978	31.7%	2,649	31.7%	
40-64	2,209	35.4%	3,117	37.3%	
65+	2,053	32.9%	2,590	31.0%	
Gender					0.941
Female	4,550	72.9%	6,108	73.1%	
Male	1,691	27.1%	2,248	26.9%	
Race/Ethnicity*					0.080
Asian	195	3.2%	146	1.8%	
Black	2,609	42.9%	3,225	38.8%	
Hispanic	3,033	49.9%	4,270	51.3	
White	239	3.9%	675	8.1%	
Employed					0.222
Yes	1,788	29.4%	2,785	33.7%	
Retired/Unable to work	2,989	49.1%	3,920	47.4%	
Other	1,314	21.6%	1,559	18.9%	
**II. Health insurance**					
Has health insurance	5,864	94.8%	7,770	93.6%	0.410
Type of health insurance					0.681
Employer	995	17.5%	1,542	20.2%	
Medicaid	2,800	49.1%	3,694	48.3%	
Medicare	1,444	25.3%	1,839	24.1%	
Other	459	8.0%	567	7.4%	
Any health insurance problems, P12M	1,160	20.7%	1,706	22.7%	0.401
If yes, type of problem					
Getting health coverage	456	8.1%	613	8.2%	0.969
Problems with billing	441	7.9%	803	10.7%	0.097
Finding affordable care	387	6.9%	512	6.8%	0.973
Navigating the systems	388	6.9%	510	6.8%	0.947
Appealing decisions	240	4.3%	317	4.2%	0.978
Another problem	178	3.2%	263	3.5%	0.759
**III. General health and behaviors**					
Self-reported general health					0.330

Good to excellent	3,350	54.0%	4,236	51.2%	
Fair/Poor	2,860	46.0%	4,039	48.8%	
Self-reported mental health					0.534
Good to excellent	4,507	72.6%	5,894	71.0%	
Fair/Poor	1,701	27.4%	2,402	29.0%	
Any physical activities, P30D	3,410	55.0%	4,783	57.6%	0.349
Health problem limits activities, past 6 months (Very limited or limited)	2,434	39.8%	3,364	40.6%	0.767
Currently smoking	1,294	20.8%	1,723	20.7%	0.971
**IV. Specific health condition**					
Ever told have asthma					0.507
Current asthma attack, P12M	755	12.1%	972	11.7%	
Asthma, no attack, P12M	955	15.4%	1,479	17.8%	
Ever told have diabetes	1,763	28.5%	2,162	26.0%	0.465
Ever told have hypertension	3,378	54.6%	4,412	53.1%	0.612
Ever told have depression	1,601	25.9%	2,139	25.9%	0.996
Have diagnosed condition/Interested in CHW program referral					
Hypertension	1,575	48.6%	---		
Diabetes	823	48.9%	---		
Asthma	435	48.9%	---		
**V. Social connectedness**					
Among 60+ years, any senior center use, P3M	654	24.4%	770	22.4%	0.578
Among all, any community center use, P3M	1,380	22.3%	1,820	22.4%	0.981
Often feel lonely or lack of companionship	1,027	16.9%	1,499	18.2	0.650

P12M, past 12 months; P30D, past 30 days; CHW, community health worker; P3M, past 3 months

Appendix Table 2. Key Themes From Open-Ended Questions, 3 Month Follow-Up: East Harlem, 2015

Key themes	Illustrative quotes
Health Advocates (HAs) provided assistance to HHAP participants in resolving health insurance issues and linking participants with medical equipment, medications, and related services	*I had Medicaid and Medicare and [the HAs] helped me combine both so that I wouldn't have to pay copayments.*<br><br>*Helped me find an insurance that directly addressed my health and solve my financial problems.*<br><br>*She resolved a problem that I was having by helping me reduce the costs that I was being charged for my prescriptions.*<br><br>*[HAs] are helping me with figuring the copays*<br><br>*I was accepted very fast [by health insurance] and did not have to travel out of my way to obtain the services I needed*<br><br>*The fact that they could fix and get my mom her wheelchair in one week when I could not get it done in a whole year.*<br><br>*Helping me with blood pressure machine.*<br><br>*They checked in on his health and helped me with problems with housing. They resolved my issues with housing.*<br><br>*Because [the HA] is making things that I didn't know available to me.*
Community health workers (CHWs) provide instrumental support to HHAP participants by linking them to needed services and helping participants engage in health promotion behavior	*Inform you of things that I didn't know about. They take my BP and weight when they visit and check in on me.*<br><br>*She showed me how to manage my knee pain, plan for my food and how to cook it, help with disability, keeping my pressure controlled, very happy with services.*<br><br>*Made me start walking, if I could give him a 20 I would give him that. I didn't like him at first but I grew to appreciate him, he pushes me and he texts me to remember about my health needs.*<br><br>*Goes with me to the appointments, eat healthy, walking, pushes me to do things.*<br><br>*She makes goals for me, she helps me with my medications and what I need to keep better, she is lovely.*

	He explaining to me about hypertension, effects of sodium, bad cholesterol, goes over foods and ingredients that are unhealthy. Very thorough with a lot go things, free yoga classes, pantry if wanted to go to, very resourceful and patient.<br><br>*Discouraged to see the doctor and appointments were being canceled and hard to reschedule appointments took months. She convinced me that I needed to go to the doctor, very good at convincing me that I need to take care of me.*<br><br>*When I showed him the problems that I am having at home in NYCHA the housing finally started to make changes in my home.*
CHWs provide and facilitate social support to HHAP participants, mitigating loneliness and social isolation, and providing a source of comfort for residents	*Plays checkers with me.*<br><br>*They really talk to you.*<br><br>*Because she listens to me and tries to help me resolve whatever issue I may have. She is there for me when I need help and is accessible over the phone.*<br><br>*A) Gets me away from drama b) voice my situation, what's going on with me c) good to have a neutral person who isn't gossipy and would spread secrets.*<br><br>*Because they brought more activities to the community center, walking to the central park, needed to gain my trust but she finally got it. As we get older we need someone to talk to, and it is very helpful.*<br><br>*He's a person that you can tell he really cares. He listens, responds, and does things to help me improve in a manner that is easy for me to understand.*<br><br>*She is concerned about my health, she is like my momma, she gets on me about health, and she feels like her care about me, she is good people.*<br><br>*You get to know that you are not the only one that has that problem. And you can end up helping somebody else.*

HHAP, Harlem Health Advocacy Partnership; CHW, community health worker; NYCHA, New York City Housing Authority
